# Control of a Production Manipulator with the Use of BCI in Conjunction with an Industrial PLC

**DOI:** 10.3390/s23073546

**Published:** 2023-03-28

**Authors:** Dmitrii Borkin, Andrea Nemethova, Martin Nemeth, Pavol Tanuska

**Affiliations:** Faculty of Materials Science and Technology in Trnava, Institute of Applied Informatics, Automation and Mechatronics, Slovak University of technology in Bratislava, 811 07 Bratislava, Slovakia

**Keywords:** EEG signal, PLC communication, disabled people

## Abstract

Research in the field of gathering and analyzing biological signals is growing. The sensors are becoming more available and more non-invasive for examining such signals, which in the past required the inconvenient acquisition of data. This was achieved mainly by the fact that biological sensors were able to be built into wearable and portable devices. The representation and analysis of EEGs (electroencephalograms) is nowadays commonly used in various application areas. The application of the use of the EEG signals to the field of automation is still an unexplored area and therefore provides opportunities for interesting research. In our research, we focused on the area of processing automation; especially the use of the EEG signals to bridge the communication between control of individual processes and a human. In this study, the real-time communication between a PLC (programmable logic controller) and BCI (brain computer interface) was investigated and described. In the future, this approach can help people with physical disabilities to control certain machines or devices and therefore it could find applicability in overcoming physical disabilities. The main contribution of the article is, that we have demonstrated the possibility of interaction between a person and a manipulator controlled by a PLC with the help of a BCI. Potentially, with the expansion of functionality, such solutions will allow a person with physical disabilities to participate in the production process.

## 1. Introduction

According to the World Health Organization records from 2011, about 15% of the world’s population lives with some kind of identified disability and about 4% of these people live with some kind of serious functioning disorders [1,2]. However, people who want to work among people with disabilities are also in high demand. Our society needs to take into account the needs of the individuals and enable them to grow personally, to develop themselves and to work. A job and regular income is a basic parameter for the inclusion of an individual in society and in in social life and also contributes to healthy mental health. According to a study [3] in the United States alone, 34.9% of people with disabilities work, compared to 76% of their non-disabled counterparts. The inability to get a job, as a rule, negatively affects the emotional and financial state of these people. For people with limited possibilities due to their disabilities, it can be helpful to fulfill their life and help them to feel like they are fully involved in ordinary society. In addition, many people with physical disorders have a relevant education. Thus, organizations do not need to train people in the processes from the very beginning, but can benefit from their technical background. For example, in 2019 in the United States, among the 38 million people at the age of 25 years and older who suffered from an identified disability, 6.7% of them possessed a bachelor’s degree [4]. According to Eurostat [5], in the European ICT market (information and communication technologies) for 2019, the shortage of specialists was 55%. In 2020, the main sector with vacant job positions was production and manufacturing. If the technical parameters and the possibility of getting decent jobs for people with limitations were met, these job positions could be filled.

One of the main problems in involving people with disabilities in the production process is the problem of their interaction with the equipment that is used in industrial environments. Therefore, to involve these people into the process, interfaces are needed to be developed, which will, on the one hand, compensate for their physical limitations, and on the other hand, allow them to interact with production equipment at the required level of functionality and safety for both production workers and the production process. The design of such an interface requires something unconventional and new. The currently used HMI (human machine interface) panels may not be enough. They require manual reactions, pressing buttons, and controlling processes by touch. This study should serve as a proof of concept, that even people with various physical disabilities can be successfully employed and can safely use various equipment in an industrial environment.

Movement is mainly controlled by the central nervous system. One of the methods of reading signals suitable for motion control is the use of non-invasive BCIs that allow for the reading of the electrical activity of the brain, namely the surface of the neocortex. For example, in Ref. [2], the authors Leila Mohammadi, Zahra Einalou, et al., investigated the possibility of cursor control using non-invasive BCI. They used the K-means clustering method to recognize the available hidden patterns in each of four modes or movements: up, down, left, and right. 

Non-invasive BCIs are now widely used and these devices allow us to read electrical potentials of different levels from different zones of the neocortex of the human brain. The surface of the neocortex is divided into four functional parts: frontal, parietal, temporal, and occipital parts [6,7,8]. BCIs’ potential is to create a new communication channel between the brain and an output device. It uses signals recorded from the human scalp, the surface of the mentioned cortex. In this way, users can control a variety of applications including, for example, simple word-processing software. In the future, BCI technology could be used as a new communication and control option for humans who cannot otherwise express their wishes or commands [9]. Therefore, essential tools like signal processing and classification methods are very necessary for improving and revealing the possibilities of BCI technologies.

In our research, we have decided to pick the BCI method out of other methods like Magnetic Resonance Imaging (MRI), Transcranial Magnetic Stimulation (TMS), Positron Emission Tomography (PET), etc., because of various factors. The BCI method is safe for the subject, because it is a non-invasive method. It is also more accessible with regard to budget and also to permissions than other methods which require special medical training.

There are many different approaches to control movement using neocortical signals. One of the approaches is based on reading signals from the motor–sensory cortex of the human brain, discussed in Ref. [10]. Movement control can also be achieved by reading signals from the occipital lobe, which is more involved in visual information processing [11]. The authors reported on the distinct time courses of cortical activation in dyslexic and control subjects during passive viewing of single words, tracked with whole-head magnetoencephalography. A striking difference was found in the left inferior temporo-occipital region, where intracranial recordings have recently identified word-specific responses within 200 msec after stimulus onset [11]. There are also combined methods that involve the processing of significant signals from different areas of the brain. For example, the Refs. [12,13] considered a combined technique based on the processing of signals from the motor–sensory and occipital cortex simultaneously. In this articles, the authors used advanced noninvasive neuroimaging techniques such as EEG and fMRI (functional magnetic resonance) that allowed them to directly observe brain activities while subjects perform various perceptual, motor, and/or cognitive tasks. The authors declared that neuroeconomics will fully reach its potential by addressing several theoretical and methodological challenges [13].

Recently, many studies have appeared that describe and classify brain activity using brain waves as possible [14]. The authors created a functional prototype that can use human EEGs to control the snake in a game with over 90% accuracy. In this experiment, two mental tasks were used to separate between turning the snake left or right: baseline (thinking nothing in particular) and mental counting [14]. A brain wave can be called an electrical map of the brain. The main types of brain waves are alpha waves, beta waves, gamma waves, delta waves, and theta waves, which are found in the ranges of 8–13 Hz, 13–30 Hz, 30–100 Hz, 0–4 Hz, and 4–8 Hz, respectively. In this article, the authors used the Emotiv Epoc device for their experiment to analyze EEG data [15]. This also confirmed that there is a huge interest in this field of study and various devices can be used for obtaining EEG data. 

The purpose of this study is to evaluate the feasibility of creating a controller for a simple manufacturing process using signals obtained using non-invasive BCI. The sensorimotor cortex is mainly responsible for the control and planning of human movement. Additionally, the task of the study is to show the possibility of controlling the production process in real time, which will allow the user to control the process without physical contact. All subjects completed this task successfully. In this study, different machine learning algorithms were used to identify patterns of brain activity corresponding to a particular command that activates specific outputs on the PLC, and subsequently the actuator itself. Four machine learning algorithms were tested (including logistic regression and several boosting algorithms), and all showed 100% accuracy in team identification under the given experimental conditions. This is due to the fact that the signals for each state/command were in different ranges and did not have cross zones, which allowed the models to correctly guess the commands after training on the training data. It should be noted that with the expansion in the range of commands, the identification accuracy may decrease.

Although the proposed solution in current state is not ready for real applications, it serves as a proof of concept for using BCI interfaces in the industrial environment. The main goal of this paper is to present the possibility and framework for identifying simple commands from the data collected via BCI and subsequently using them to control industrial equipment. 

With a sufficient expansion in the range of commands and a decrease in the reaction speed of the actuators, it is possible to use the system in real production, at least for two purposes:

(1) Control of the actuator or part of the production process or part of the conveyor without physical contact in manual mode.

(2) Testing the functionality of the selected actuator or part of the production process to identify problems encountered during operation.

These two tasks could potentially be solved by people with physical disabilities using the BCI and PLC interfaces connected to each other [16,17].

## 2. Materials and Methods

As a part of the experiment, we were determined individual signals for a simple command for a PLC station using two types of sensors; the first data detection circuit with the frontal motor–sensory cortex of the brain and the second perception circuit, which in itself also had sensors, to receive signals from the occipital and temporal lobes. Based on the received signals, different machine learning models were obtained, after which the accuracy of their predictions was compared in the model testing test.

### 2.1. Scheme of Experiment

**Figure 1:**
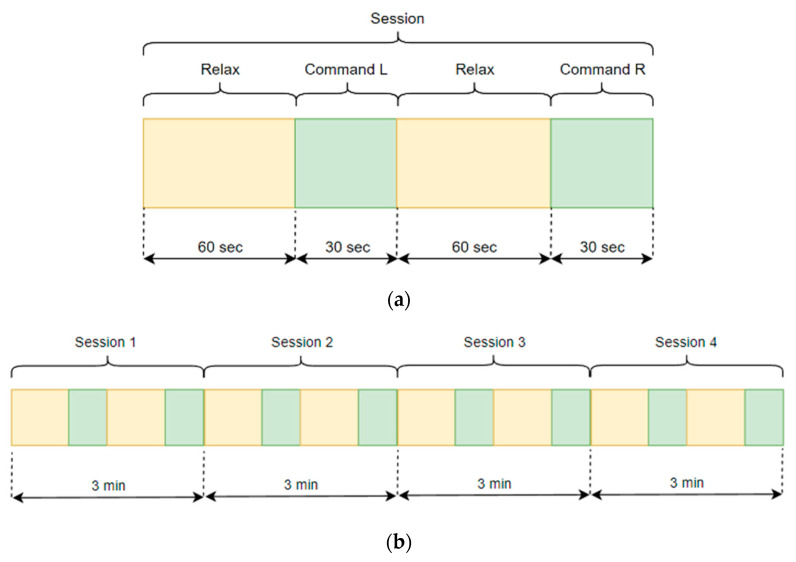
Description of the time process for (**a**) one experiment; (**b**) all sessions.

As subjects for our experiment, four healthy men aged 30 to 50 years were selected. It should be noted, however, that for a deeper test of universal patterns of work, the subjects should be selected to represent different sexes, ages, and physical conditions. Our subjects signed an agreement on the provision of biometric signals and their processing for scientific purposes. Figure 1 shows the scheme of the experiment. 

**Figure 2:**
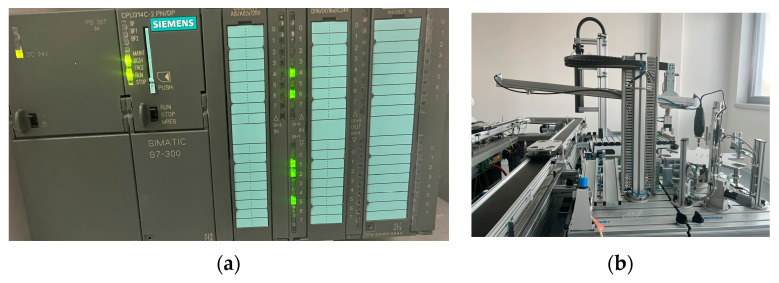

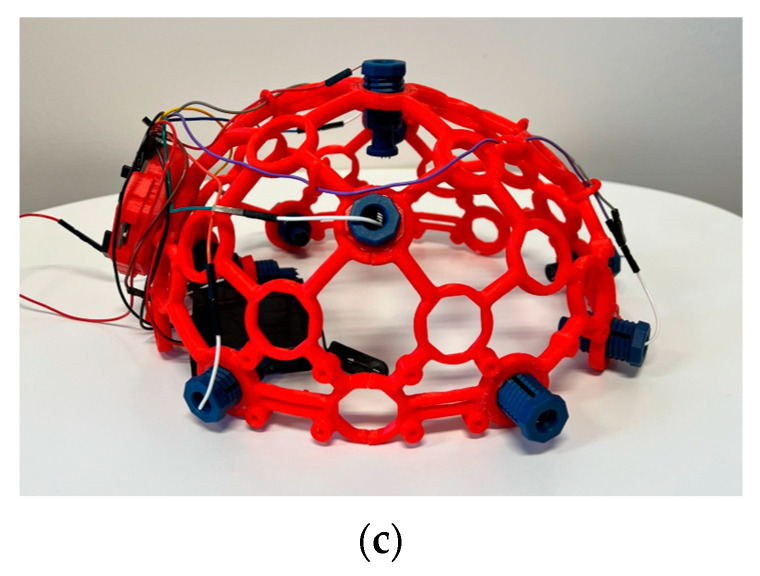
(**a**) Programmable Logic Controller (S7-314, Siemens), (**b**) an actuator in which we control two positions, movement to the right and to the left, (**c**) EEG headset (OpenBCI).

In the first stage after connecting the equipment, the subject is at rest for 1 min and does not try to give any commands, see in Figure 2. After that, the subject mentally focuses special attention on the left hand for 30 s. Then, the subject takes another break for 1 min. After that, the subject focuses his attention on the right hand for 30 s. The total time for one experiment is 3 min. To check the reproducibility of the experiment, each subject is conducted in four sessions. To avoid possible defects such as eye movement or body movement, the subject sits with his eyes closed, relaxed, in a comfortable sitting position. In addition, the experiment was supervised. During each session there was a supervisor constantly checking that the participant was not physically moving, which would corrupt the experiment itself. All experiments were held in the first half of the day where the subjects were not tired and had good energy. 

**Figure 3:**
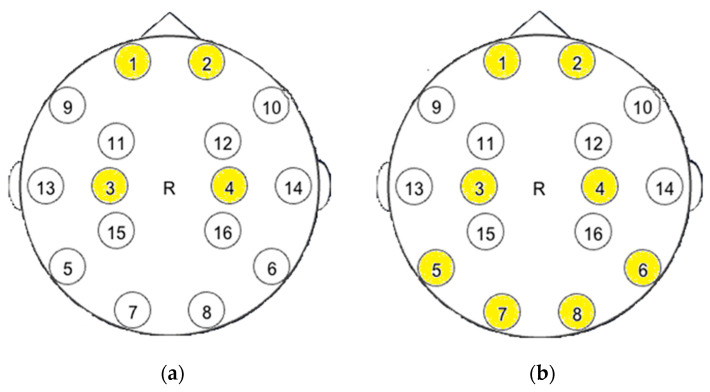
Scheme of the location of the electrodes: (**a**) frontal, parietal; (**b**) frontal, parietal, occipital, temporal. Yellow are nodes that were available for our version of BCI headset.

Figure 3 shows the layout of the electrodes whose data were used to train our model. In the first case, data from electrodes 1, 2, 3, and 4 were used, which corresponds to the location of electrodes Fp1, Fp2, C3, and C4 along the international system 10–20. In the second case, data from electrodes 5, 6, 7 and 8 were added, which corresponds to electrodes P7, P8, O1, and O2. According to the 10–20 system, each electrode position is designated by a letter to identify the lobe of the human brain and another letter or number to identify the location of the electrode within the lobe. In this case, and for purposes of our experiment, the received data came from the frontal (Fp1, Fp2), central (C3, C4), parietal (P7, P8), and occipital (O1, O2) electrodes.

According to the standard international 10–20 system, each electrode is designated by a letter and a number, where the letter represents a region of the cerebral cortex: frontal (F), central (C), parietal (P), occipital (O), temporal (T) and fronto-parietal (FP). Even numbers are used for the right hemisphere of the brain, odd numbers for the left hemisphere. In general, the 10–20 system provides 21 electrodes proportionally distributed over the scalp, but this version of Open BCI had only 8 sensors as shown in Table 1. The location of the sensors was chosen in such a way that all sensors were located in different areas of the brain. Table 1 explains the function of the electrodes for each area of the brain. This work aims to identify the most discriminant electrode channels in terms of accuracy metrics. Fp1 and Fp2 are located in areas that are responsible for attention, judgment and impulse control. C3 and C4 are in the sensorimotor cortex, which is responsible, among other things, for movement planning. P7 and P8 are in the parietal lobe near the back of the head, where spatial information is processed. O1 and O2 are located in the occipital lobe, which processes visual information.

### 2.2. Equipment Description

**Figure 4:**
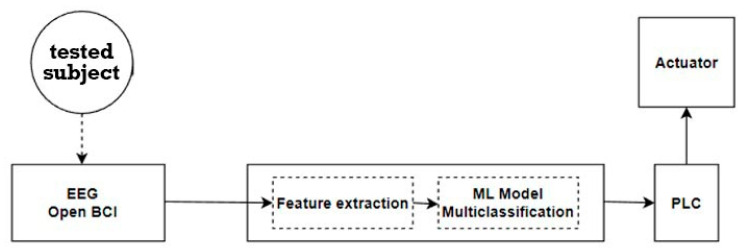
Flow chart of the experiment with all of its parts.

Figure 4 shows the general equipment connection diagram. An OpenBCI helmet© was installed on the head of each participant of the experiment, and it was checked that each electrode had contact with the surface of the head (if necessary, each electrode was adjusted separately).

The OpenBCI interface communicates with the computer via Bluetooth. On the PC, the signal is passed through a pre-trained ML model, after which, using the SNAP-7 library, the result is sent to the PLC S7-314 to one of the controller outputs. PLC outputs are connected directly to the actuator to control its movement.

The OpenBCI kit consists of an Ultracortex Mark IV EEG headset, a board for receiving and processing signals from biosensors on eight channels, an OpenBCI Cyton Board, and a programmable dongle OpenBCI (for Bluetooth communication) see in Table 2 and Table 3.

The actuator shown in Figure 2b has two degrees of freedom, as well as a grip for moving objects, but within the framework of the experiment, the actuator is controlled only along the horizontal axis. During the control period, the manipulator either moves to the left side, is stationary, or moves to the right side, depending on the command sent by the participant in the experiment.

### 2.3. Data Acquisition

Data collection in this case was different from what one would expect from traditional data collection methods. In our research, we used OpenBCI Cyton Board for human EEG acquisition. We have eight available passive Ag/AgCl electrodes which were placed at the locations: POz, PO3, PO4, PO7, PO8, O1, O2, and Oz according to the standard international 10–20 system. There were also reference and ground electrodes. These electrodes were placed on the mastoid bony surfaces behind the right and left ear. The obtained signals were sampled at 250 Hz during the experiment. Another feature to mention that was used during all experiments is that the electrode–skin impedance was kept just below 10 kΩ.

## 3. Results

**Figure 5:**
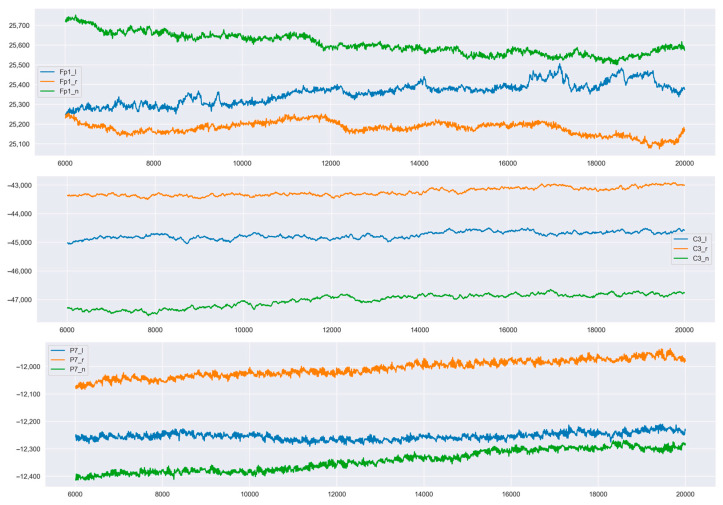

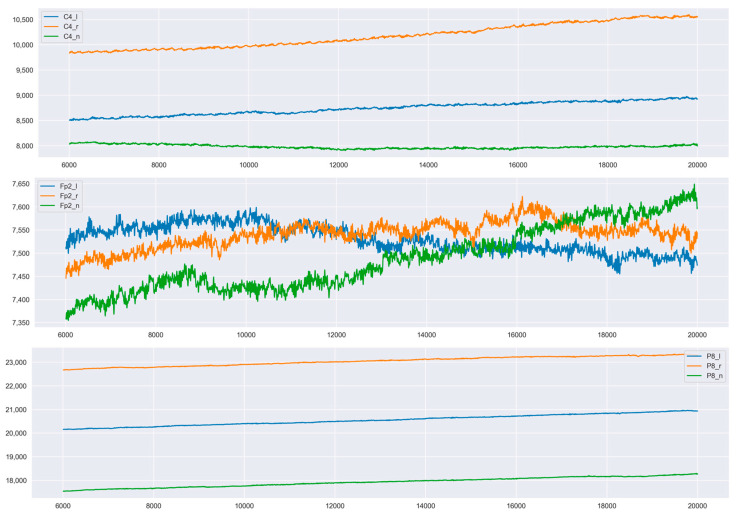
Changes in signals for one of the subjects for three states (rest, movement to the left, movement to the right) over time. Orange represents left hand, blue represents right hand, and green represents neutral state of mind.

**Figure 6:**
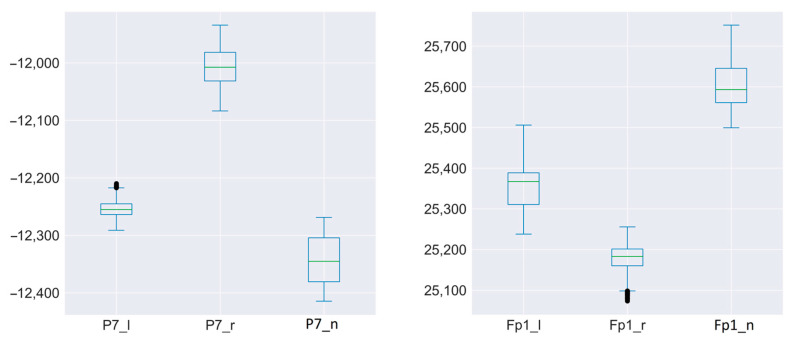

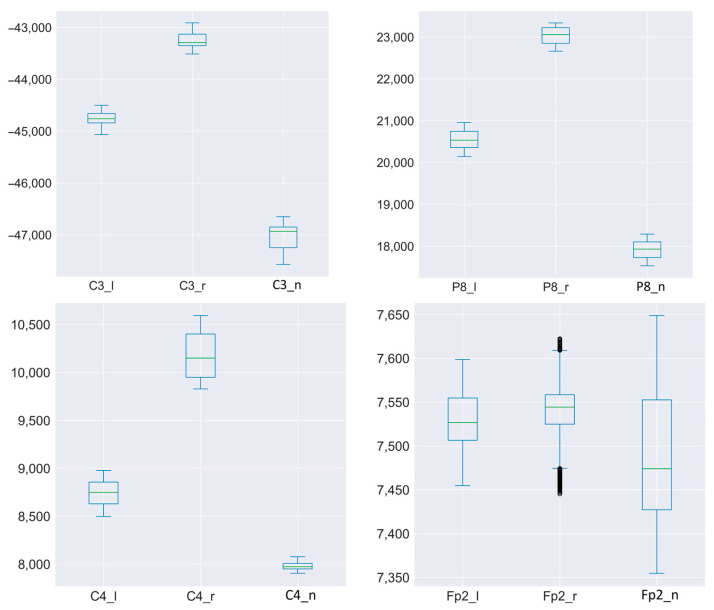
Distribution of signal magnitude for one of the subjects for three states (rest, movement to the left, movement to the right).

Figure 5 and Figure 6 show the three channels for six sensors of one of the subjects. Fp1, Fp2—frontal lobe left and right. C4—near the parietal lobe. P7, P8—occipital lobe closer to the midline. 02—occipital part.

For each signal from each sensor, the same time interval was taken. Thus, it was possible to superimpose signals for different states from one sensor on one graph. It can be seen that the signals for different states (left, right, rest) have intersection areas for only one sensor.

The states from the FP2 sensor, which was located on the front right side of the neocortex, had the least variability. It is difficult to say what exactly is the reason for such a reaction in the right frontal lobe (this reaction requires additional research).

The sensors did not change their location during the full session of the experiment. It is clear from the graphs that the focus on different teams or states was different. Significant intersection of signals occurred only in one case: when receiving signals from the FP2 sensor.

A clear separation of signals can be traced in all subjects, although the levels of signals from each subject differed.

**Figure 7:**
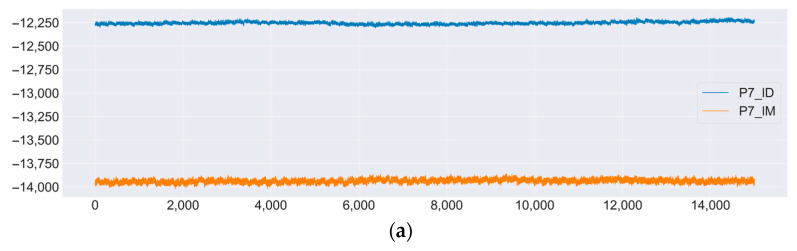

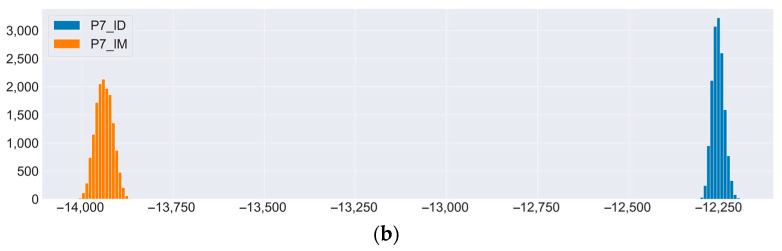
Signals from sensor P7 for two participants. (**a**) Signal changes over time. (**b**) Distribution of values for each of the signals. It is clearly seen that for the P7_IM signal, all values were grouped in the −14,000 microvolt region, while for the P7_ID signal, these values were in the −12,250 microvolt region.

In Figure 7, there is a comparison of the signals for two subjects for the same commands from the same sensors. As can be seen from the graphs, for the two participants in the experiment, there was a clear separation for each type of signal.

## 4. Discussion

Based on BCI, a simple drive control system via PLC was developed. All participants were able to successfully control the drive after pre-training the model on individual user data (Supplementary Materials). Additionally, all subjects were able to quickly enter a state of rest before the experiment. However, in the future, one of the subjects, when concentrating on one of the commands, involuntarily set in motion the left or right hand. Thanks to visual control, it was possible to establish this and conduct a repeated series of experiments in order to exclude involuntary physical movements.

During the experiment, several tasks were recorded, the study of which is possible in future experiments.

Although the data received from the sensors clearly identified each command, the signal levels were different for each user. In this case, it was necessary for to collect individual data for each participant in the experiment and retrain the model in order to accurately identify the commands. The difference in signals can be associated both with the individual characteristics of the participant, and with a small difference in the location of the sensors relative to the neocortex zones. More precise positioning of sensors is discussed in Refs. [18,19].

Additionally, the problem of different signal intensities can be associated with different emotional and physical states of the participants. In future experiments, it is possible to additionally control the state of the participant by measuring their pulse during the experiment, as was done in [20,21]. This study looked at the relationship between resting heart rate variability and performance with the P300-BCI.

Secondly, despite the successful results of the experiment, the control of the actuator was not fully implemented. The drive has two degrees of freedom. There is also an invader feature. Extending the drive control with these functions will allow us to talk about the full control of the drive (for manual control mode). Modern BCI systems using SSVEP [16] can contain up to 15 commands. However, for hybrid BCIs using P300 and SSVEP, more than 100 command codes have been implemented [17]. The question also arises about the long-term use of such control, because in total, each session was no longer than 12 min. It is possible that as the session grows and the number of possible commands increases, the accuracy and speed of switching between commands will deteriorate.

## 5. Conclusions

This study was devoted to the design and implementation of using EEG signals for control systems for robotic arm control. We have focused on building the bridge between human biosignals and machine control. 

In this study, a control system for a simple production arm using BCI and PLC was designed and tested. All subjects were able to successfully operate the manipulator, although individual system settings were required for each subject. Mastering the necessary commands for control was quite easy, and none of the subjects had problems focusing on one of the three states. Breaks between two key states, according to the subjects, made it is easier to focus on the task following the relaxation phase.

However, in order for the system to potentially be used in production, it is necessary to reduce the break time between commands for the actuator. Additionally, it is necessary to expand the range of commands so that they cover the full range of possible actions of the manipulator. In addition, the system should be more versatile in terms of reconfiguration for each new user, i.e., reconfiguration time should be reduced to a minimum or, if possible, this process should be avoided altogether.

Finally, it is necessary to conduct testing with persons with impaired motor functions, since they are the intended end users of the system.

## Figures and Tables

**Table 1:** Hardware specification of OpenBCI 32 bit Board (Open BCI, Brooklyn, New York, NY, USA).

Specifications	-
Input channels	8
Microcontroller	PIC32MX250F128B
Data resolution	24-bit channel
Programmable gain	1, 2, 4, 6, 8, 12, 24
Digital operating voltage	3.3 V
Analog operating voltage	+/−2.5 V
Input voltage	~3.3–12 V
Converter	Texas Instruments ADS1299 ADC

**Table 2:** Hardware specification of OpenBCI Dongle.

Specifications	-
Radio module	RFD22301
USB to serial converter	FT231X

**Table 3:** Hardware specification of Siemens Simatic S7-300.

Specifications	-
CPU	314C-2PN/DP Compact
Work memory	192 KB
DI	24
DO	16
AI	4
AO	2
Interface	2 Ethernet PROFINET

## Data Availability

Not applicable.

## Supplementary Materials

The following supporting information can be downloaded at: https://www.mdpi.com/article/10.3390/s23073546/s1, Figure S1: Denoised data example for one participant, sensor FP2. Orange: thoughts on right hand, Blue: thoughts on left hand. Figure S2: Data example with noise for one participant, sensor FP2. Orange: thoughts on right hand, Blue: thoughts on left hand. Figure S3: Data plot for second participant, sensor FP1. Orange: thoughts on right hand, Blue: thoughts on left hand. Figure S4: Data plot for second participant, sensor FP2. Orange: thoughts on right hand, Blue: thoughts on left hand. Figure S5: Data plot for second participant, sensor C4. Orange: thoughts on right hand, Blue: thoughts on left hand. Figure S6: Data plot for second participant, sensor P8. Orange: thoughts on right hand, Blue: thoughts on left hand. Figure S7: Data plot for second participant, sensor P7. Orange: thoughts on right hand, Blue: thoughts on left hand. Figure S8: Data plot for third participant, sensor FP1. Orange: thoughts on right hand, Blue: thoughts on left hand. Figure S9: Data plot for third participant, sensor FP2. Orange: thoughts on right hand, Blue: thoughts on left hand. Figure S10: Data plot for second participant, sensor C4. Orange: thoughts on right hand, Blue: thoughts on left hand. Figure S11: Data plot for second participant, sensor P7. Orange: thoughts on right hand, Blue: thoughts on left hand. Figure S12: Data plot for second participant, sensor P8. Orange: thoughts on right hand, Blue: thoughts on left hand. Figure S13: Data plot for forth participant, sensor FP1. Orange: thoughts on right hand, Blue: thoughts on left hand. Figure S14: Data plot for forth participant, sensor FP1. Orange: thoughts on right hand, Blue: thoughts on left hand. Figure S15: Data plot for forth participant, sensor C4. Orange: thoughts on right hand, Blue: thoughts on left hand. Figure S16: Data plot for second participant, sensor P7. Orange: thoughts on right hand, Blue: thoughts on left hand. Figure S17: Data plot for second participant, sensor P8. Orange: thoughts on right hand, Blue: thoughts on left hand.
