# Supplementary material for: Control of a Production Manipulator with the Use of BCI in Conjunction with an Industrial PLC
Source: Sensors (Basel). 2023 Mar 28;23(7):3546. doi: 10.3390/s23073546 (PMC10098813; doi:10.3390/s23073546)
Supplement: Supplementary file 1 [file sensors-23-03546-s001.zip › sensors-2236396-supplementary.pdf]

Attachement with data set for all participants

Denoised

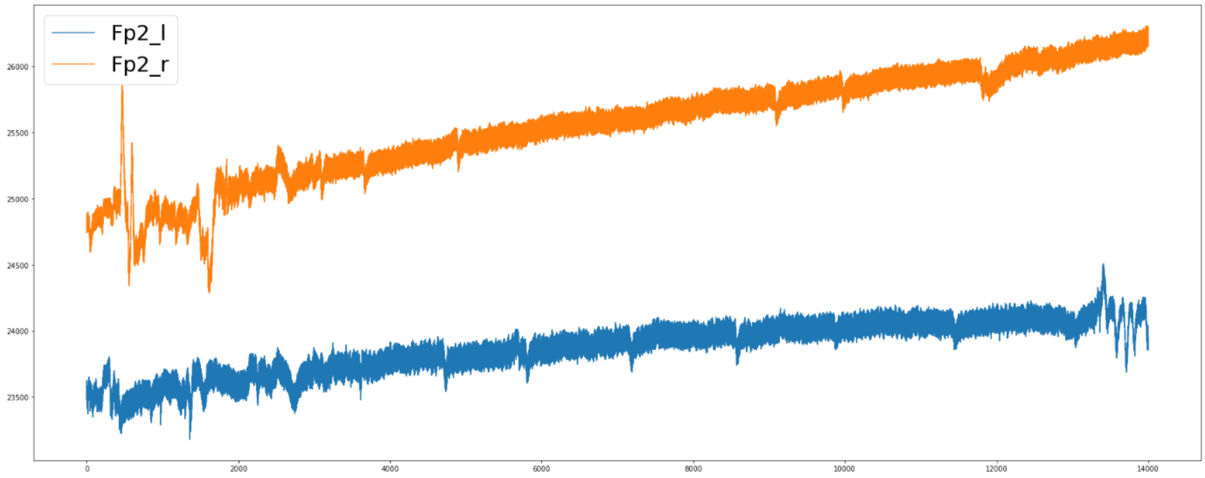

Figure S1: Denoised data example for one participant, sensor FP2. Orange: thoughts on right hand, Blue: thoughts on left hand.

With noise:

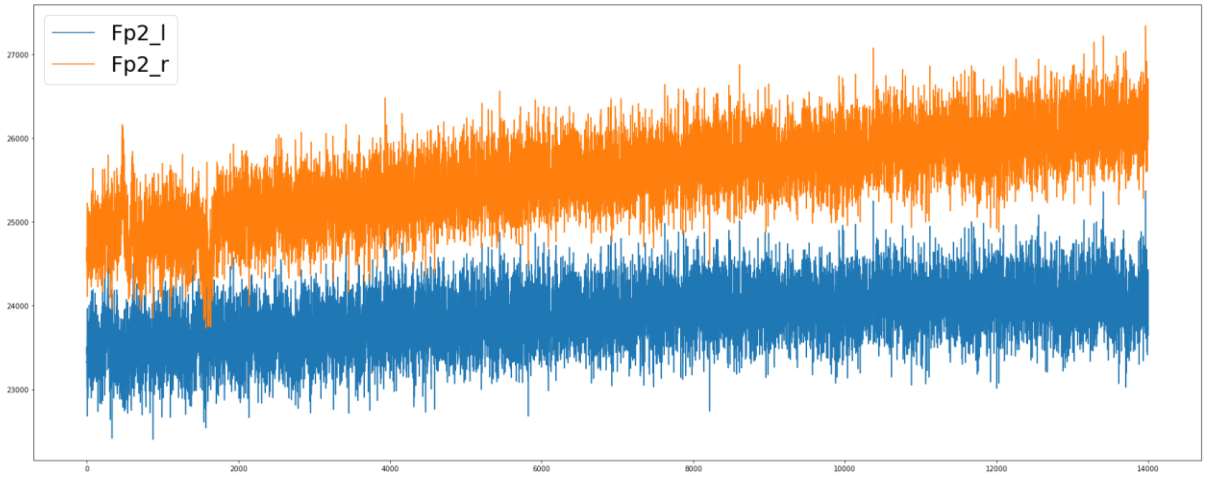

Figure S2: Data example with noise for one participant, sensor FP2. Orange: thoughts on right hand, Blue: thoughts on left hand.

For second participant:

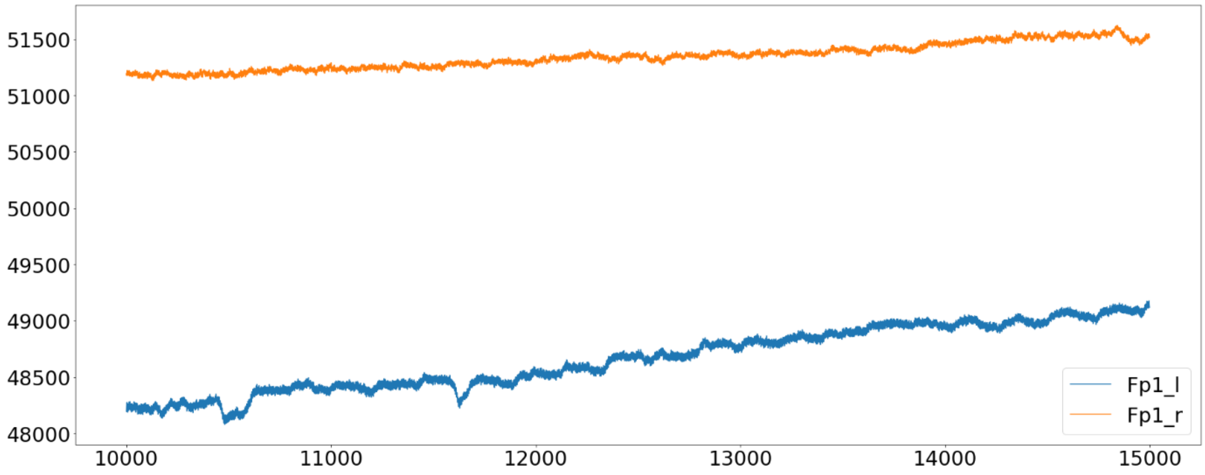

Figure S3: Data plot for second participant, sensor FP1. Orange: thoughts on right hand, Blue: thoughts on left hand.

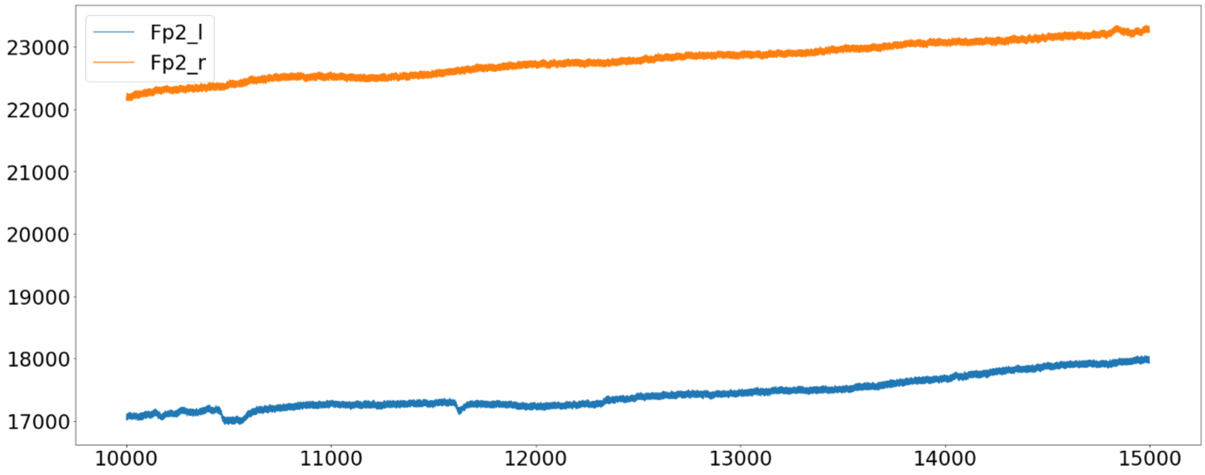

Figure S4: Data plot for second participant, sensor FP2. Orange: thoughts on right hand, Blue: thoughts on left hand.

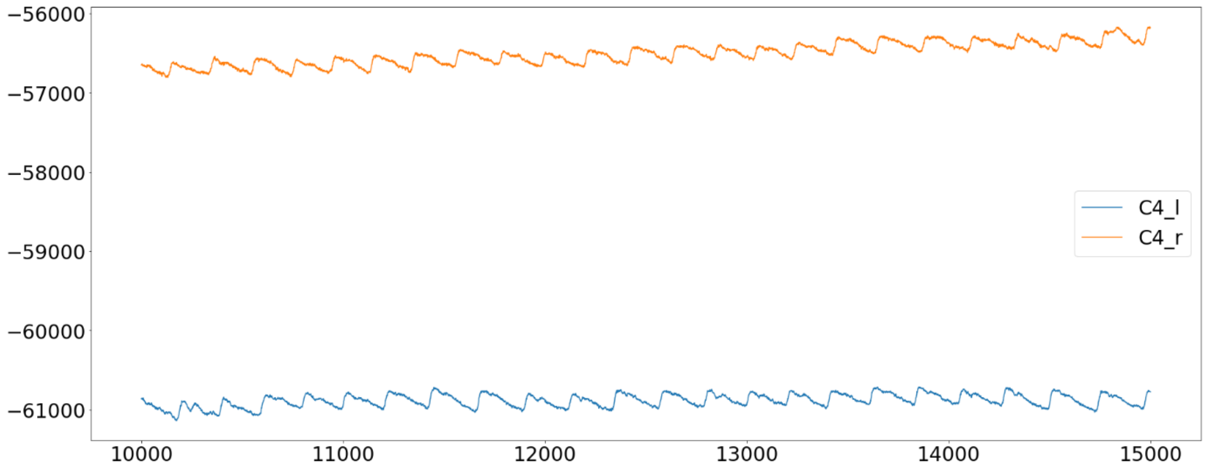

Figure S5: Data plot for second participant, sensor C4. Orange: thoughts on right hand, Blue: thoughts on left hand.

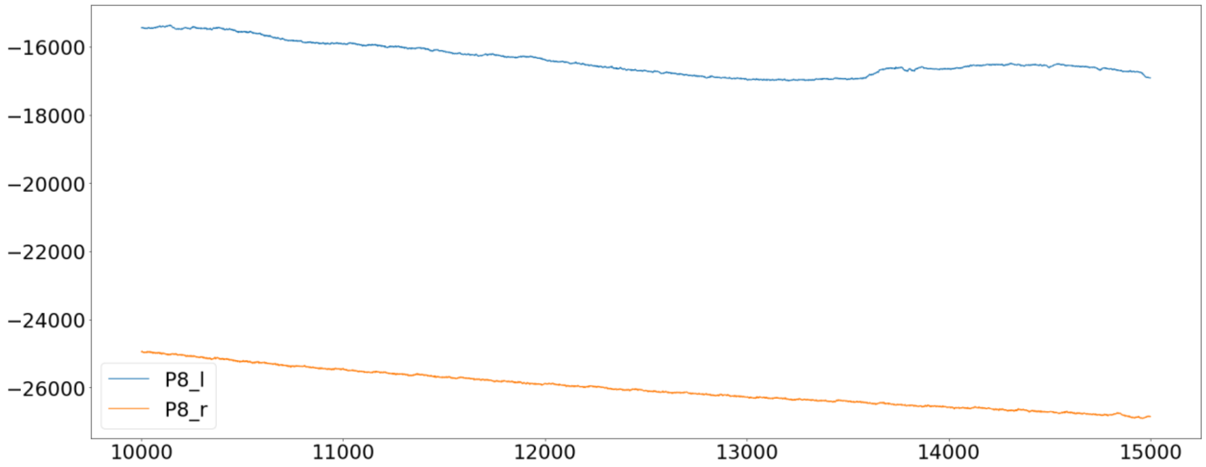

Figure S6: Data plot for second participant, sensor P8. Orange: thoughts on right hand, Blue: thoughts on left hand.

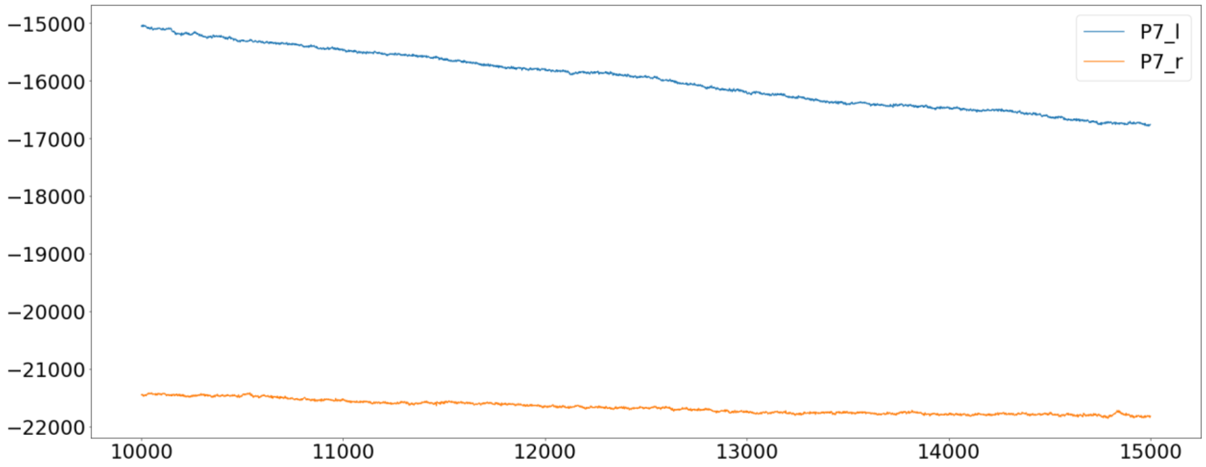

Figure S7: Data plot for second participant, sensor P7. Orange: thoughts on right hand, Blue: thoughts on left hand.

For third participant:

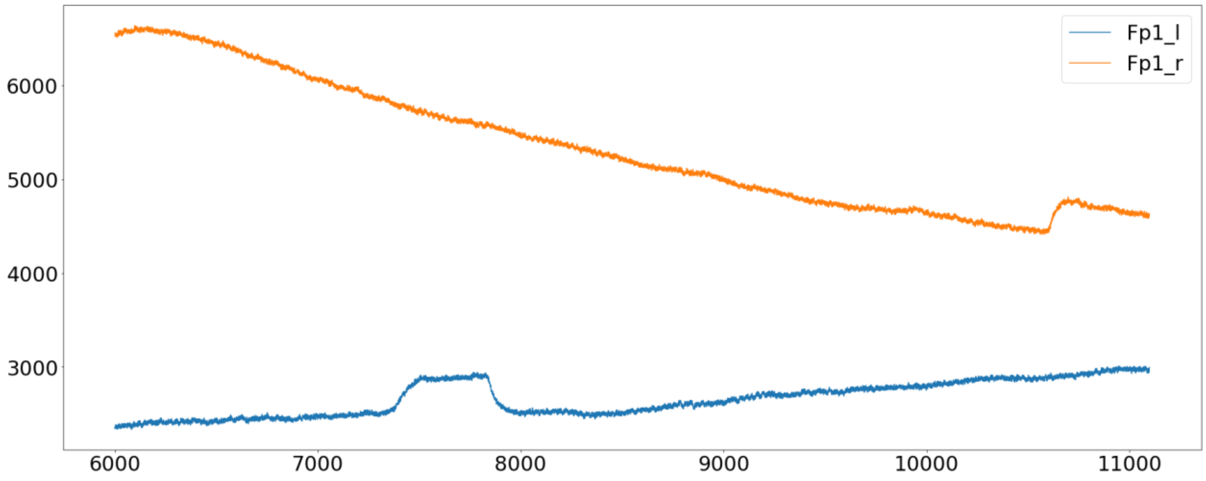

Figure S8: Data plot for third participant, sensor FP1. Orange: thoughts on right hand, Blue: thoughts on left hand.

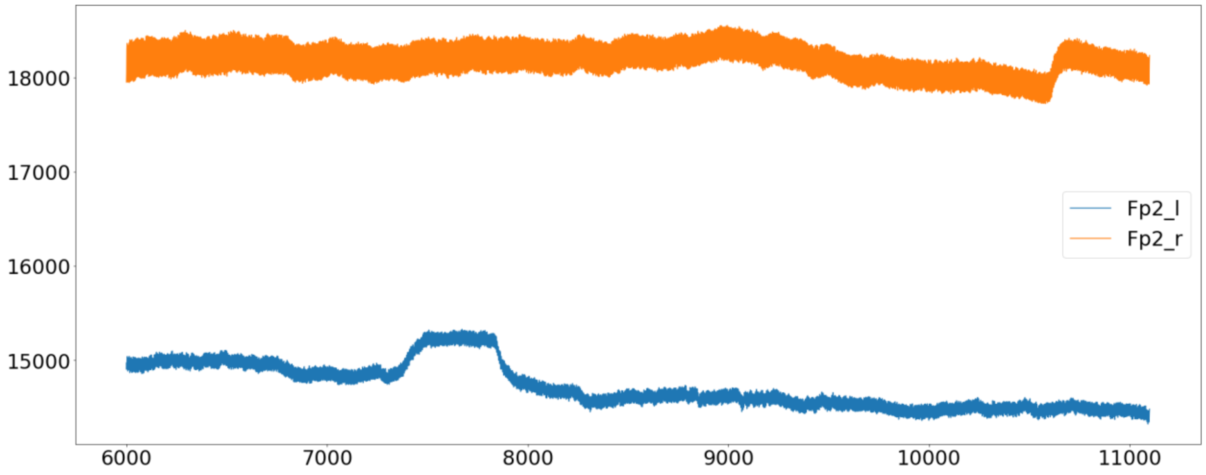

Figure S9: Data plot for third participant, sensor FP2. Orange: thoughts on right hand, Blue: thoughts on left hand.

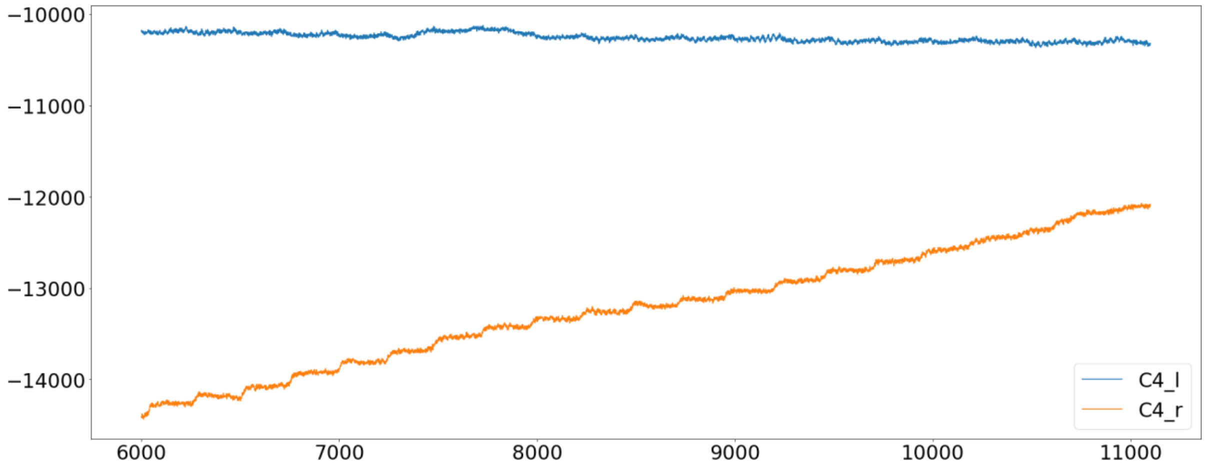

Figure S10: Data plot for second participant, sensor C4. Orange: thoughts on right hand, Blue: thoughts on left hand.

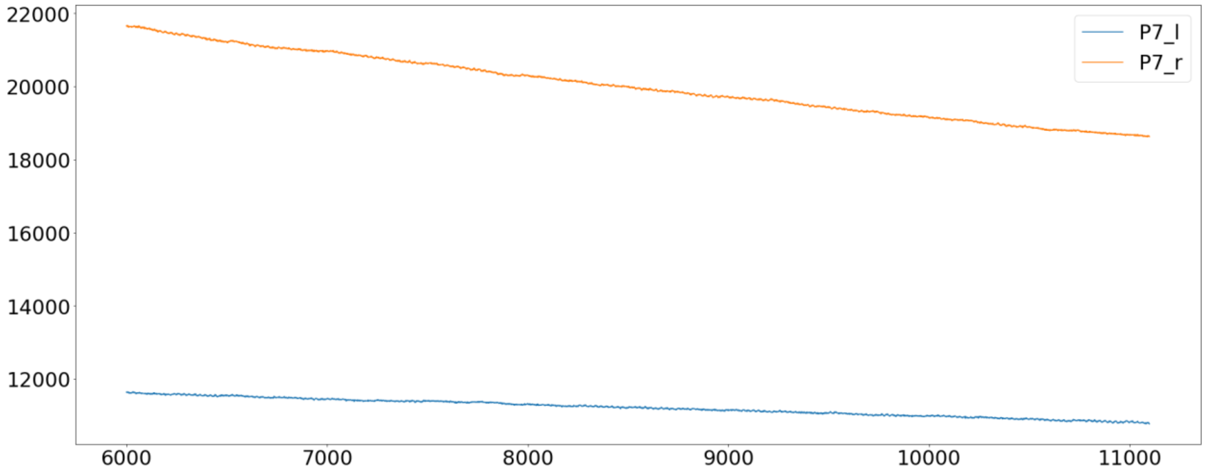

Figure S11: Data plot for second participant, sensor P7. Orange: thoughts on right hand, Blue: thoughts on left hand.

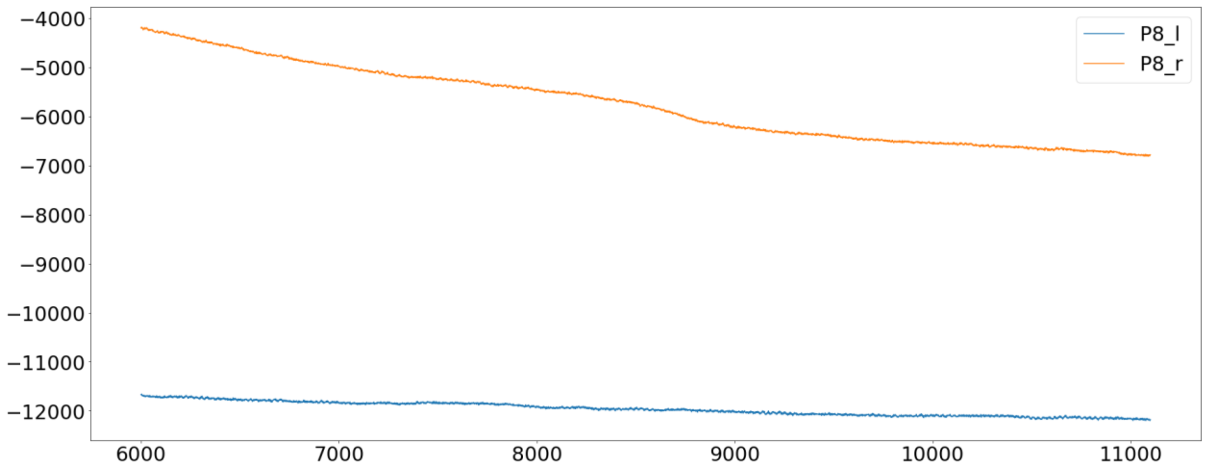

Figure S12: Data plot for second participant, sensor P8. Orange: thoughts on right hand, Blue: thoughts on left hand.

For forth participants:

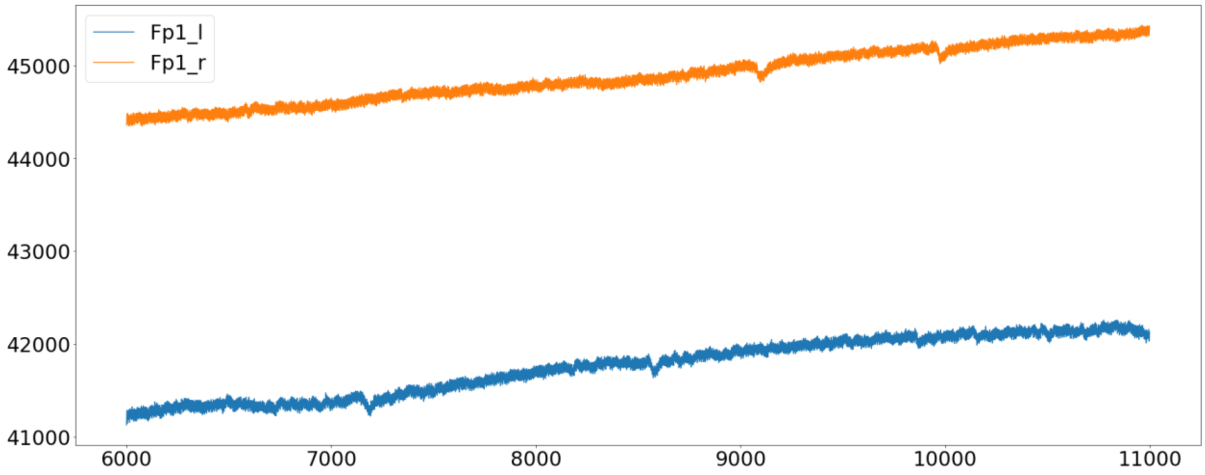

Figure S13: Data plot for forth participant, sensor FP1. Orange: thoughts on right hand, Blue: thoughts on left hand.

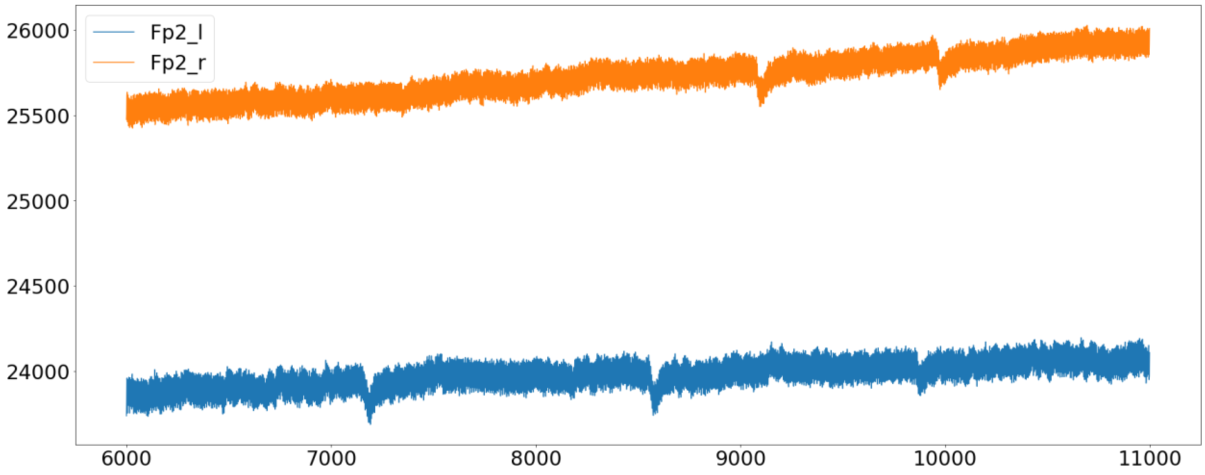

Figure S14: Data plot for forth participant, sensor FP1. Orange: thoughts on right hand, Blue: thoughts on left hand.

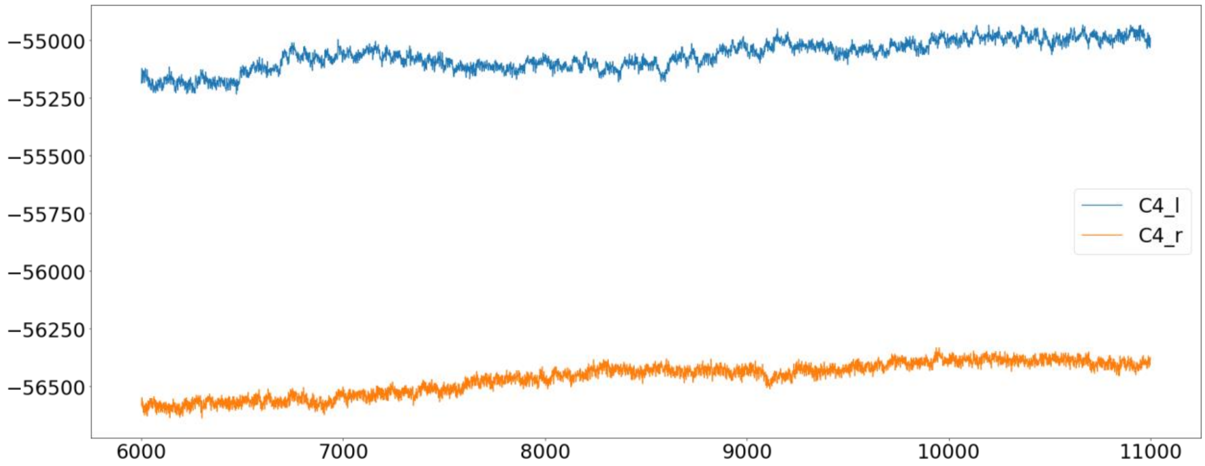

Figure S15: Data plot for forth participant, sensor C4. Orange: thoughts on right hand, Blue: thoughts on left hand.

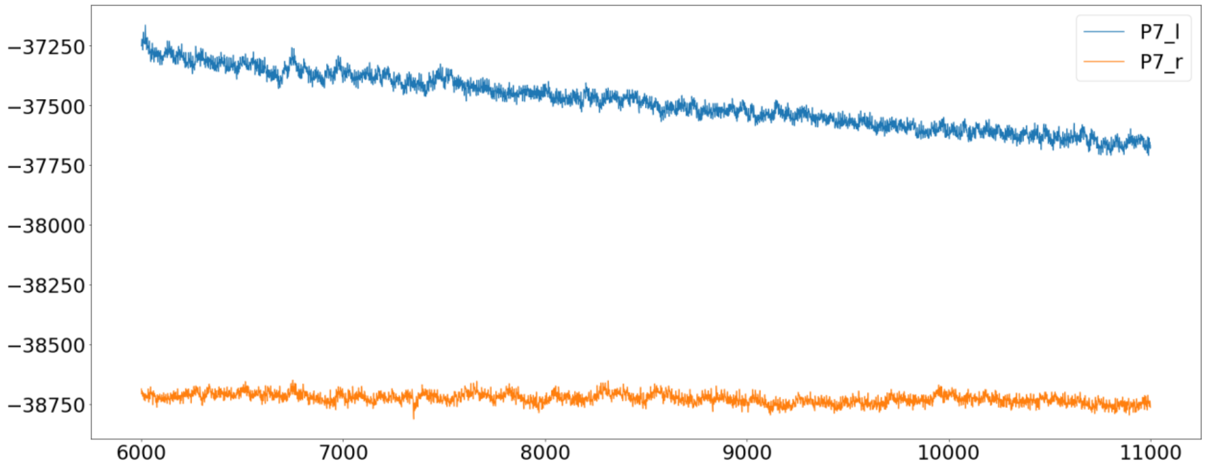

Figure S16: Data plot for second participant, sensor P7. Orange: thoughts on right hand, Blue: thoughts on left hand.

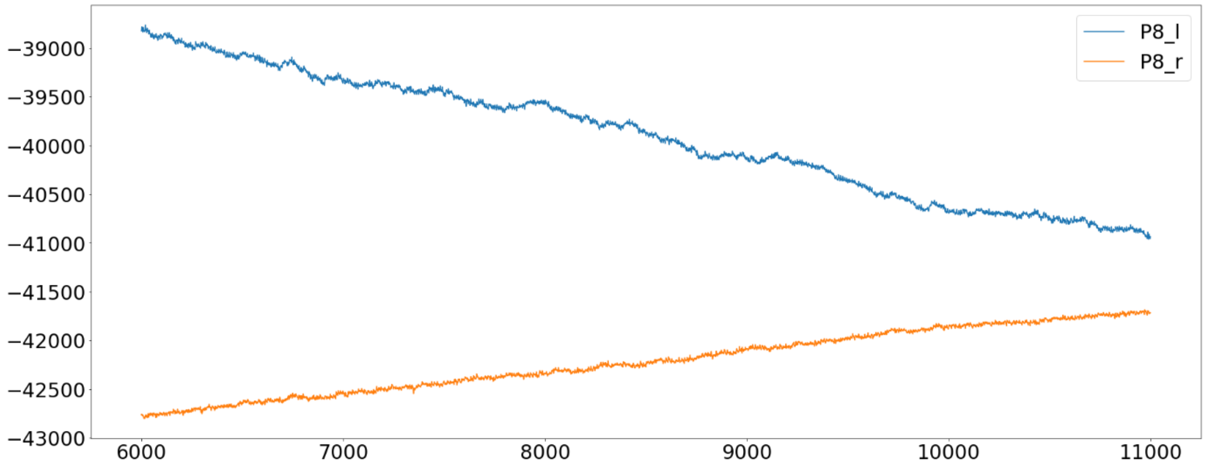

Figure S17: Data plot for second participant, sensor P8. Orange: thoughts on right hand, Blue: thoughts on left hand.
